# A Bevel Gear Quality Inspection System Based on Multi-Camera Vision Technology

**DOI:** 10.3390/s16091364

**Published:** 2016-08-25

**Authors:** Ruiling Liu, Dexing Zhong, Hongqiang Lyu, Jiuqiang Han

**Affiliations:** School of Electronic and Information Engineering, Xi’an Jiaotong University, Xi’an 710049, China; bell@xjtu.edu.cn (D.Z.); hongqianglv@xjtu.edu.cn (H.L.); jqhan@xjtu.edu.cn (J.H.)

**Keywords:** multi-camera vision, bevel gear, defect detection, dimension measurement

## Abstract

Surface defect detection and dimension measurement of automotive bevel gears by manual inspection are costly, inefficient, low speed and low accuracy. In order to solve these problems, a synthetic bevel gear quality inspection system based on multi-camera vision technology is developed. The system can detect surface defects and measure gear dimensions simultaneously. Three efficient algorithms named Neighborhood Average Difference (NAD), Circle Approximation Method (CAM) and Fast Rotation-Position (FRP) are proposed. The system can detect knock damage, cracks, scratches, dents, gibbosity or repeated cutting of the spline, etc. The smallest detectable defect is 0.4 mm × 0.4 mm and the precision of dimension measurement is about 40–50 μm. One inspection process takes no more than 1.3 s. Both precision and speed meet the requirements of real-time online inspection in bevel gear production.

## 1. Introduction

The bevel gear, also called the automobile shift gear, is an important part in an automobile transmission. Its quality directly affects the transmission system and the running state of the whole vehicle [1]. According to statistics, about one third of automobile chassis faults are transmission faults, in which gear problems account for the largest proportion [2]. Although the bevel gear’s design, material and manufacturing technique are quite mature and standard, in today’s high-speed production lines there are still inevitably defects such as dimension deviations, surface scratches, crushing, dents or insufficient filling, etc. Defective gears will not only reduce the performance of the car, but also cause security risks and potentially huge losses to manufacturers [3].

**Figure 1:**
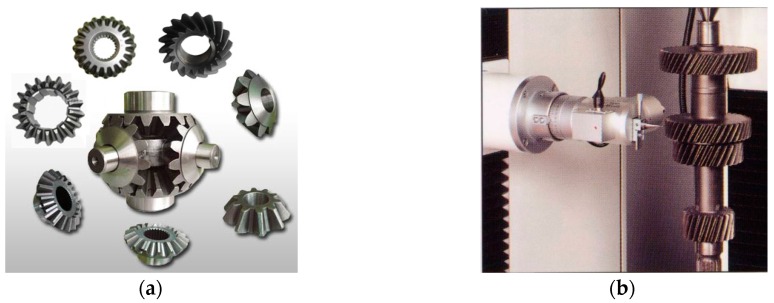
(**a**) Samples of bevel gear; (**b**) Gear measuring instrument of Osaka Seimitsu Precision Machinery Corporation.

Figure 1a shows some kinds of bevel gears. The shape and structure of bevel gears are complicated and the types of defects various, so bevel gear quality inspection is very difficult. At present it is mainly completed manually, which is tedious and inefficient. It is hard to guarantee the quality stability and consistency. Recently Osaka Seimitsu Precision Machinery Corporation in Japan developed a high precision gear measuring instrument as shown in Figure 1b, but the inspection speed is very slow and cannot meet the requirements of high-speed production lines. There has been gear defect detection technology based on machine vision, but it’s mainly used in the inspection of broken teeth and flat surfaces of general gears [4]. There is no precedent research for bevel gears with complicated shape and structure [5]. Because of the multiple measurement tasks and diverse defects, the traditional single vision system cannot obtain enough information. To measure the multiple dimensions quickly and accurately, to detect and classify the defects completely and precisely, a more efficient vision system is needed.

Machine vision is such an advanced quality inspection technology which uses intelligent cameras and computers to detect, identify, judge and measure objects [6,7,8,9,10,11,12,13,14]. To solve the above problems, this paper develops a multi-vision bevel gear quality inspection device which combines defect detection and dimension measurement in one system. Three efficient image processing algorithms named Neighborhood Average Difference (NAD), Circle Approximation Method (CAM) and Fast Rotation-Position (FRP) are proposed. The system successfully solves the problem of bevel gear defect detection and measurement at high speed and accuracy. One can find a short Chinese article describing this system in [15].

## 2. System Design

### 2.1. Inspection Project and Requirement Analysis

**Figure 2:**
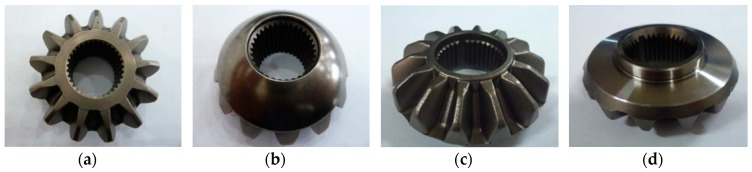
Samples of gear A_4_ and F_6_: (**a**) Top surface of gear A_4_; (**b**) Bottom surface of gear A_4_; (**c**) Top surface of gear F_6_; (**d**) Bottom surface of gear F_6_.

The defect detection and dimension measurement system developed in this paper is designed for two specific types of bevel gears, A_4_ and F_6_, as shown in Figure 2.

**Figure 3:**
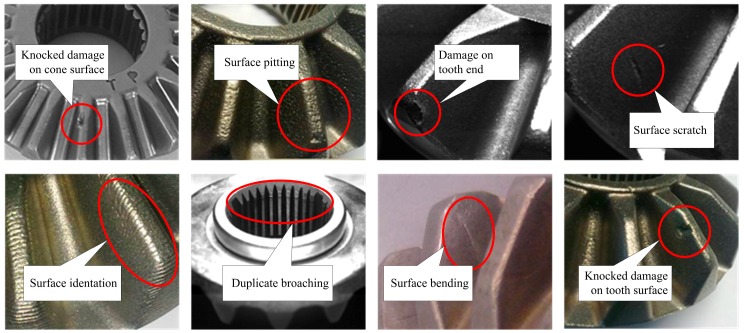
Examples of defects on bevel gear surface.

The requirements of defect detection and measurement are as follows:

(1) Defect detection of tooth end surface, spherical surface and mounting surface, including knock damage, cracks, insufficient filling, folding, scratches, dents or gibbosity, repeated spline broaching, etc. As shown in Figure 3. Defects greater than 0.5 mm × 0.5 mm should be detected.
(2) Measurement of bevel gear’s height, diameters of two circumcircles, spline’s big and small diameters, end rabbet’s diameter. Measurement accuracy is 80–100 μm.
(3) Defect detection speed is about 1 s for each surface.

### 2.2. Hardware Design

Bevel gears have many teeth and each tooth has different lateral surfaces. For example, gear A_4_ has 13 teeth and 26 surfaces; gear F_6_ has 14 teeth and 28 surfaces. The defects are diverse. How to realize the real-time online inspection of all the teeth and surfaces is a big challenge. One approach is using a single camera and rotational shooting mode, but it requires a high-precision rotary positioning system. The inspection speed is too low to meet the requirements. Because the bevel gear’s teeth surfaces are uniformly distributed and rotationally symmetric, we propose a solution which can complete image acquisition and processing synchronously by using multiple high-speed cameras. The speed was an order of magnitude faster than single camera rotary positioning system.

**Figure 4:**
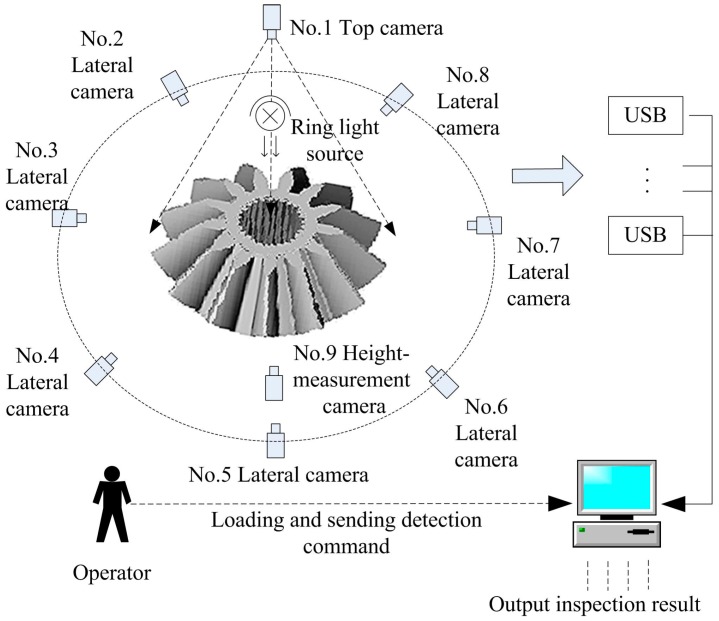
Diagram of bevel gear inspection system.

Figure 4 shows the total plan of our inspection system. Nine high speed USB cameras are used to capture different parts of the bevel gear simultaneously. The top camera is the core of the whole image acquisition system. It undertakes the positioning of the bevel gear, defect detection and measurement of the top and bottom surfaces.

Seven side-view cameras are emplaced evenly around the bevel gear, with a 45° tilt angle with respect to the horizontal plane and perpendicular to gear tooth groove, detecting the lateral teeth surfaces omni-directionally. In addition, one more camera with a horizontal view is located on the platform to measure the bevel gear’s height. After the gear is loaded, the operator gives a command that all of the cameras acquire images at the same time. An industrial computer receives and processes the images one by one and gives control signals according to the detected results. To meet the different accuracy requirements of bevel gear defect detection, three types of industrial cameras are adopted in this system. They are 3 megapixels, 2 megapixels and 1.3 megapixels, respectively. We can calculate that one pixel represents 40–50 μm according to the gear’s actual size and image size. That is to say, the accuracy in the bevel gear’s height and diameter measurements is 40–50 μm.

**Figure 5:**
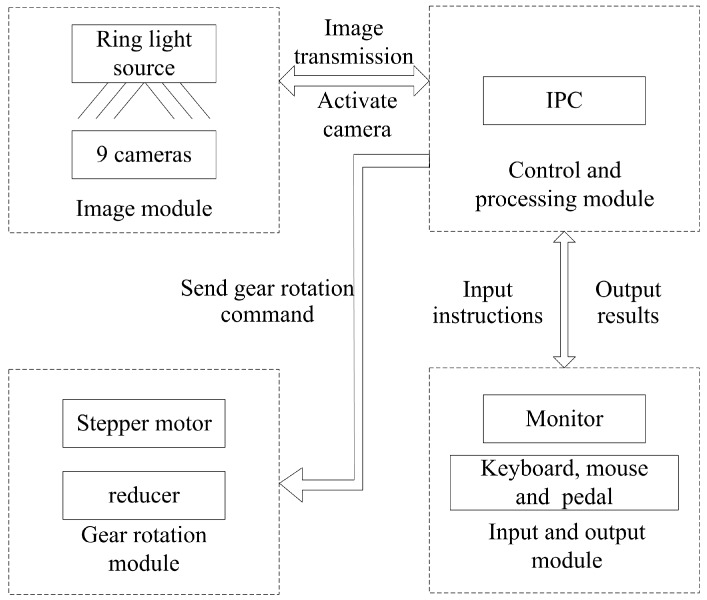
Hardware structure of the bevel gear inspection system.

The system hardware includes four modules, which are camera module, control and process module, I/O module and gear rotary module, as shown in Figure 5. The camera module consists of nine high speed industrial cameras and a top ring light. The ring light illuminates the whole system. The cameras capture every part of the bevel gear. The control and processing module is responsible for image processing and output of instructions and inspection results. I/O module includes monitor, keyboard, mouse and pedal. The monitor displays the results. The software is operated by mouse and keyboard. The foot pedal starts the inspection of each surface. Software button starter and automatically trigger are also provided in this system. The gear rotation module is made up of a stepping motor and a reducer. It rotates the bevel gear precisely in the defect detection of special parts and template acquisition section.

**Figure 6:**
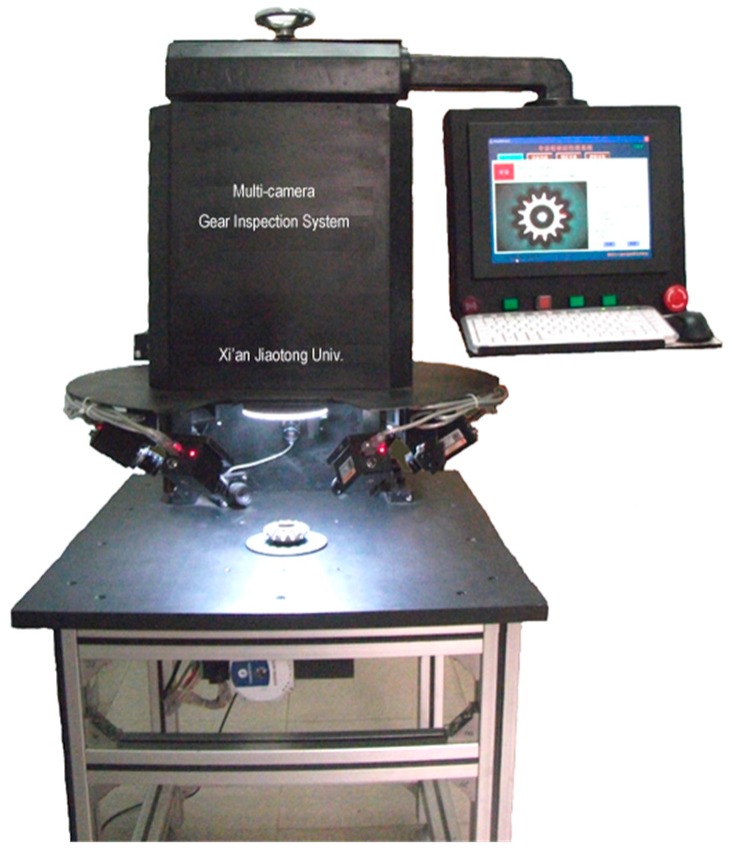
Prototype of multi-camera bevel gear inspection system.

Figure 6 shows the prototype of this multi-camera bevel gear quality inspection system. In order to adapt to various inspection tasks and objects, a series of position adjusting elements are designed. The height of top camera and illumination can be adjusted. Each camera can be adjusted slightly in the horizontal direction. For side-view cameras, the tilt angle and distance to the gear are adjustable in a large range, so the system can detect and measure the vast majority of bevel gears and other gears or parts of similar structure.

### 2.3. Software Design

**Figure 7:**
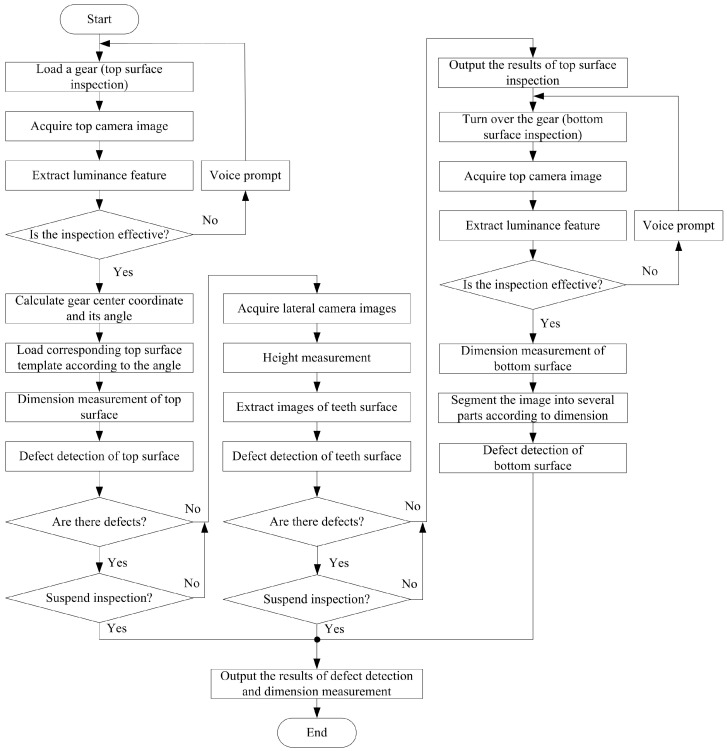
Software flowchart of bevel gear inspection system.

Based on above hardware, a fast and multifunctional bevel gear inspection software is developed under the MFC and OpenCV platforms. The inspection includes two steps, top and bottom surface respectively. The software can automatically identify the current inspection is the top or the bottom of a bevel gear, and whether the bevel gear is in the right place. Top inspection includes two sub-modules, top surface and lateral surface, each one compose of size measurement and defect detection. The software flowchart is shown in Figure 7.

**Figure 8:**
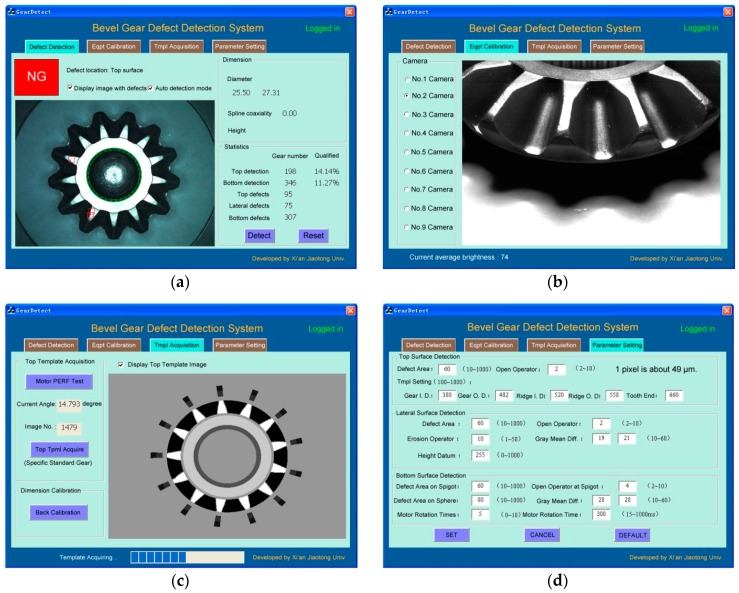
Soft interface of bevel gear inspection system: (**a**) Defect detection; (**b**) Equipment calibration; (**c**) Template acquisition; (**d**) Parameter setting.

For a new inspection task, the software runs in order of top→lateral→bottom surface inspection. If any fault is found, the program marks it at once and outputs the defects and size measurement results, and then terminates. The software of the bevel gear inspection system employs a multi-thread technique. It includes four modules, which are defect detection, equipment calibration, template acquisition and parameter setting. The equipment calibration module calibrates the position, apertures and focuses of the nine cameras. The template acquisition module completes the sample collection and size calibration of a standard gear. The parameter setting module adjusts the related parameters for different kinds of inspection tasks. The defect detection module marks the faults in the gear image, and shows all the size and statistical data. Figure 8 shows the appearance of the software.

## 3. Key Algorithms in Defect Detection

Because the defects of the bevel gears are complex and various, and the inspection time is short, the general algorithms cannot meet the requirements. In this system, we use multi-threading technology and propose several efficient image processing algorithms, which are NAD, CAM and FRP. The following introduces these key algorithms in detail.

### 3.1. Neighborhood Average Difference Method

From the point of texture, all kinds of defects are shown as waves, twists, undulations or roughness of the surface, thus defects can be detected by analyzing gear texture. Texture description operators based on gray-scale histograms include average, standard deviation, smoothness, third order moment, consistency and entropy, etc. A large number of experiments show that the combination of third order moment and smoothness do best in bevel gear defect detection, but these operators are sensitive to outside interference. The threshold setting is complicated and based on massive numbers of experiments and the results cannot show the characteristics of defects directly and comprehensively (such as area). In our system, we present an algorithm called Neighborhood Average Difference (NAD), which can extract the defects entirely and define larger ones as faults, ignoring tiny ones. The steps are as follows:

*Step 1*: Count the number of nonzero pixels in a neighborhood, denoted by *N*, given the neighborhood range is *L* × *L* pixels, here *L* = 30.
*Step 2*: Calculate the sum of gray values in the neighborhood: $S=\overset{L-1}{\underset{i=0}{\sum }}\overset{L-1}{\underset{j=0}{\sum }}p\left(i,j\right)$, in which *p*(*i,j*) is the pixel value of point (*i,j*).
*Step 3*: Calculate the average pixel value in a neighborhood: *M = S/N*.
*Step 4*: For each point *p*(*i,j*) in the neighborhood, if |*p*(*i,j*) − *M*| > *δ*, *δ* is a threshold, then the point is marked by setting *p*(*i,j*) = 255.
*Step 5*: Scan the marked points in the image. If they are in connected region and the size of the region is greater than a threshold, then the region is a defect. Single marked points and tiny connected regions are ignored.

In addition, interference such as the impurities, dust and dirt on the surface can be removed partly by image dilation and erosion processing.

### 3.2. Circle Approximation Method

**Figure 9:**
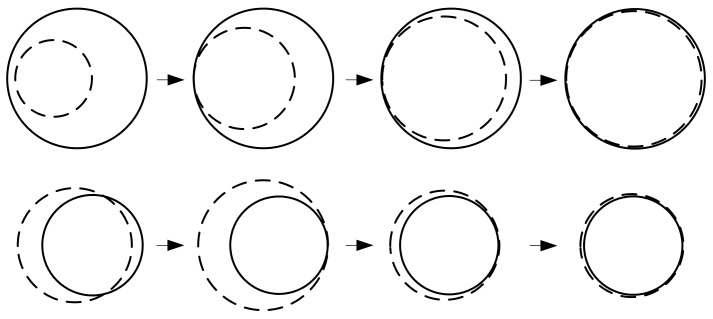
Circle Approximation Method: Each row shows an example of circle fitting process. The solid circles are to be detected and the dash circles are regulated to match the solid ones.

Circle detection is a classical problem in image processing [16,17,18,19]. To detect the actual and virtual circles on the bevel gear fast and accurately, an algorithm named CAM is presented. The main idea is: Draw a circle with initialized center and radius, then expands or contracts it slowly and moves the center continuously until it is tangent to the circle to be detected. Then keep adjusting its radius and center on condition that they remain tangent. The drawn circle will move closer and closer to the circle to be detected until they match perfectly. Figure 9 shows this process.

Circle center, radius and rotation angle step are three initial inputs of this method. The center and radius can be approximated only asking the drawn and the target circles have some common area. The more accurate the initial parameters are, the faster the method gets an idea result. It utilizes the positioning result in dimension measurement, and the steps are as follows:

*Step 1*: Extract the edge of the circle (actual or virtual) to be detected by threshold segmentation of the original image. Store the edge in target pixel set *C*.
*Step 2*: Set the initial center (*x*_0_, *y*_0_) and radius *r*_0_ of the drawn circle. Set the rotation angle step *θ*.
*Step 3*: Increase or decrease radius *r*_0_, *r*_0_ = *r*_0_ + *δ_r_*, or *r*_0_ = *r*_0_ − *δ_r_*, *δ_r_* is the change value in each adjustment.
*Step 4*: Calculate the pixel set *C’* on the drawn circle with the current center and radius. Find the intersection of *C* and *C’*, record as *X*. If *X* is empty, return to Step 3.
*Step 5*: If *X* is non-empty and the number of its elements is *X_num_*, judge whether *X_num_* satisfies the matching condition. If satisfies, output the center and radius information, otherwise move the center in its eight neighborhoods and find the position where *X* has the least elements, record and return to step 3.

**Figure 10:**
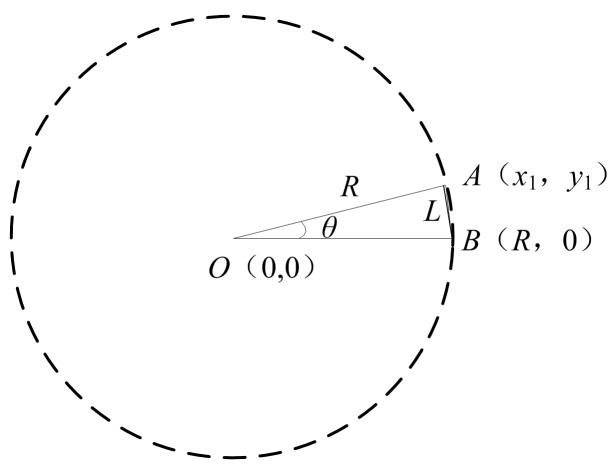
The calculation of rotation angle step *θ* in CAM.

The parameter *θ* is used in calculation of set *X*. Figure 10 shows how it is defined. *R* is the radius. *A* and *B* are the starting and ending point of a rotation. Line segment *L* connects *A* and *B*. For computational convenience, the center coordinate is set to (0, 0), *B* is set to (*R*, 0), *A* is set to (*x*_1_, *y*_1_), then:

(1) $$x_{1}=R\cos\theta ,\;y_{1}=R\sin\theta$$

(2) $$L^{2}={\left(R-R\cos\theta \right)}^{2}+R^{2}\sin^{2}\theta$$

(3) $$\theta =\arccos[(2R^{2}-L^{2})/2R^{2}]$$

Because the image is discrete, *L* ≥ 1, then across $\left(\frac{2R^{2}-1}{2R^{2}}\right)\leq \theta \leq \pi$.

The method counts target pixels on the drawn circle based on the parameter *θ*, it searches step by step like the second hand of a clock. The value of *θ* is closely related to the cost of computation. Theoretically, the greater the *θ* is, the faster the algorithm is, but the robustness of the algorithm will be decreased and the detection accuracy will be reduced at the same time, so the detection accuracy, the execution speed and the robustness of the algorithm should be all taken into account to set the angle step *θ*. In addition, *θ* can be set dynamically. In early of the algorithm a bigger *θ* is used to approach the detected circle quickly. *θ* becomes smaller as soon as the drawn circle touches the detected circle which is then searched and fitted in a small range. The algorithm is efficient and accurate with a dynamic theta.

Similar to Hough Transform, our method is robust to image noise and disturbance in virtue of cumulative sum. It has a simple searching idea and only requires a preprocessing of threshold segmentation. While in Hough Transform, preprocessing of edge detection or skeleton extraction is very time consuming.

**Figure 11:**
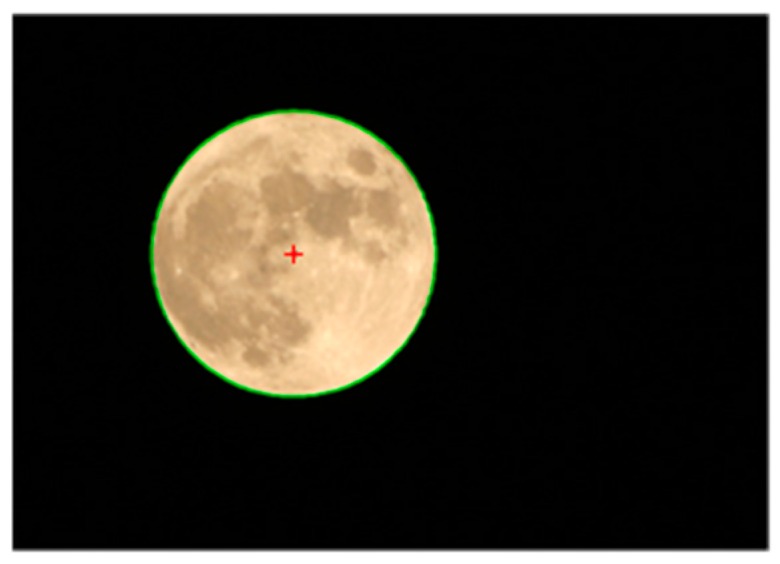
Moon picture (952 × 672 pixels) and circle fitting result. Red cross is the center.

In order to verify the detection results of our algorithm, we compare it with Hough transform by fitting the moon in Figure 11. The results of the two methods are almost identical and cannot be distinguished by naked eye. Details of the comparison are shown in Table 1. The data show that CAM is about 50 times faster than Hough Transform, with little difference in initial parameter setting. In addition, the classical Hough Transform cannot be used in high resolution images because the parameter space matrix will increase dramatically with the increase of the image size, resulting in a large amount of computation and storage space. However, our circle approximation algorithm is not restricted in this aspect. It is simple and fast with less computation, and is very suitable for the high precision circle detection situation.

CAM is suitable for fitting circles when a good initialization is available. Hough is suitable for fitting circles in the presence of noise and multiple models (multiple circle arcs). A solution from Hough transform will generally require refinement using an iterative method like CAM.

Our method is more suitable for detection of circles which have been positioned approximately. In actually, with the auxiliary system composed by sensors and cameras, the location of bevel gear won’t deviate greatly. The gross location of the circle can be determined by software in advance. So our method can be successfully applied to most of the industrial circle detection occasions. The detection and production efficiency can be improved significantly with its speed advantage.

**Figure 12:**
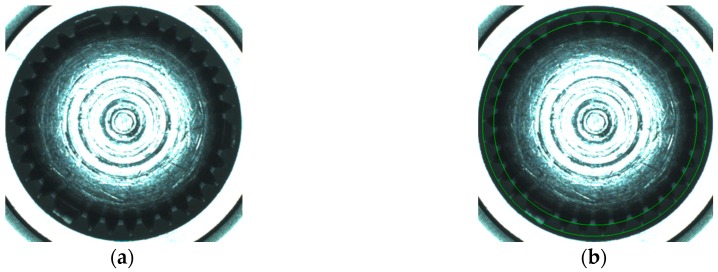
Fitting the spline circles by CAM: (**a**) Image of spline; (**b**) Detection results of inscribed and circumscribed circles of spline (denoted by green circles).

Like other circle detection methods, our method can detect solid circles and arcs. But it can also detect virtual circles such as gear spline circle, inscribed circle and circumscribed circle of a polygon. Figure 12 shows the fitting results of spline virtual circles on gear F_6_ by our method.

### 3.3. Fast Rotation-Position

**Figure 13:**
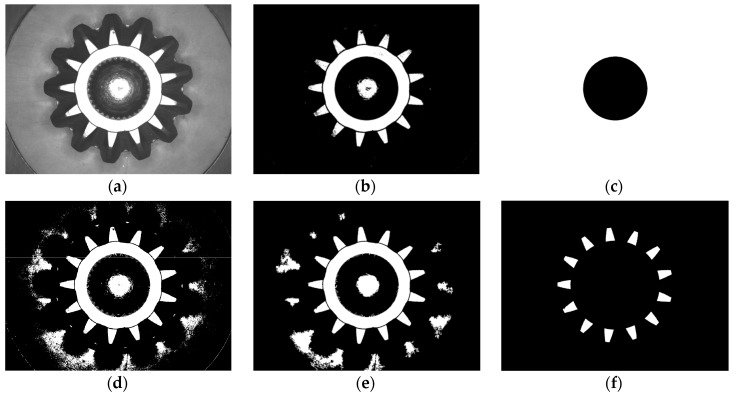
The image preprocess of FRP method: (**a**) Top surface image; (**b**) Segmentation for inner circle (with a larger threshold); (**c**) Inner circle detected; (**d**) Segmentation for teeth end (with a smaller threshold); (**e**) Result of area filting; (**f**) Teeth end image.

In order to carry out accurate inspection of the bevel gear, the position of the workpiece should be determined quickly after loading. It includes horizontal, vertical and rotational position. This system uses a mechanical device to realize the horizontal and vertical position. Due to the complex gear structure and the short feeding time, it is difficult to achieve effective rotary position by hardware. We solve this problem by image processing method. Taking into account the center rotary symmetry of the bevel gear, the rotation position is divided into three steps which are center positioning, image acquisition of tooth surface and the rotary angle calculation:

(1) Center positioning. To cut out the image of the inner circle, first segment the top camera image with a certain threshold, then remove interference of impurity and inherent defects by foreground and background area filting. The circle center and diameter are extracted by area statistics and coordinates averaging. Figure 13a–c show this process.
(2) Teeth-end image extraction. Segment Figure 13a again with a smaller threshold value and cut out the entire teeth-end image. Remove interference of impurity and inherent defects by foreground and background area filtering. Then draw two circles with the center calculated above and proper radiuses, extract only the teeth-end surface, as shown in Figure 13d–f.
(3) Calculate the rotation angle. In order to implement template matching algorithm in defect detection, the original image of the bevel gear should be transformed to a standard position to extract the matched temple. The following shows how to calculate the rotary angle α.

**Figure 14:**
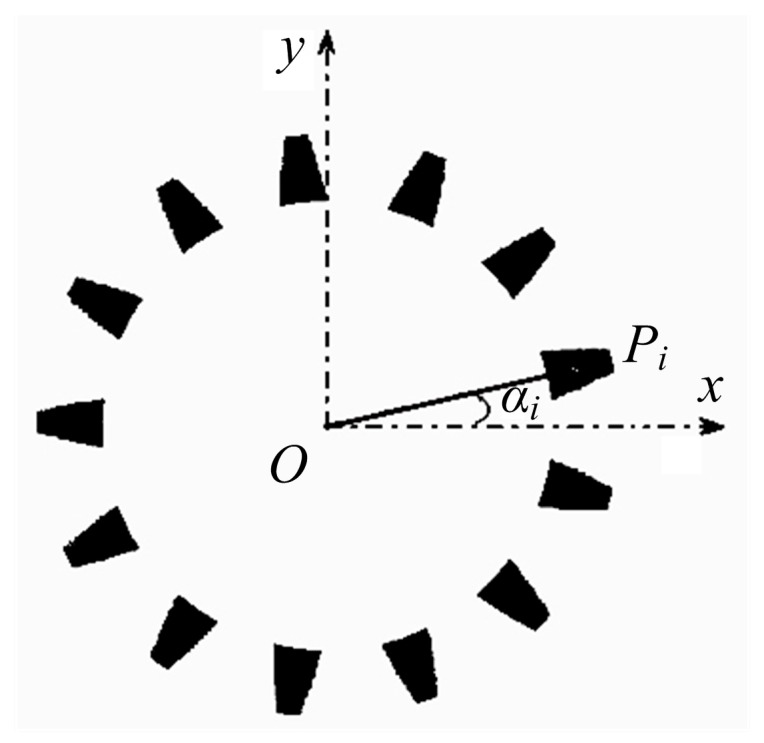
Calculation of gear rotation angle.

For example, bevel gear A_4_ has 13 teeth, which are distributed evenly. The angle between two teeth is 360°/13 = 27.69°, as shown in Figure 14. *O* is the center of the gear and *P_i_* is the geometric center of a tooth. The angle between *x*-axis and line *OP_i_* is defined as *α_i_*. For each tooth, *α_i_* is converted into the first quadrant and within 0°–27.69°. For gear A_4_, *α_i_* = *α*_1_, *α*_1_,…, *α*_13_. We eliminate any *α_i_* that has great difference with others, and calculate the mean of the rest, which is defined as the rotation angle *α* of the gear to be detected. Experiments show that the rotation-position method provides excellent accuracy. The error is about 0.005°, which can meet the requirements of the gear position determination.

### 3.4. Template Matching and Collection

In machine vision, we often compare an input image with a standard image to find the difference or target, which is called template matching. It’s fast and efficient. In this system we use template matching a lot to extract the area to be detected, eliminating the interference of non-inspection areas and improving the inspection speed.

**Figure 15:**
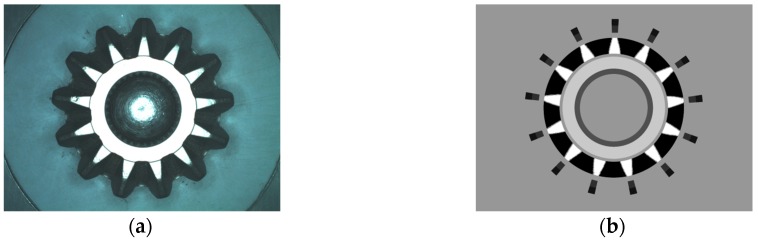
(**a**) Top surface of gear A_4_; (**b**) The matching template.

Figure 15a is the top surface image of bevel gear A_4_ and Figure 15b is the matching template. In Figure 15b, the value of background and edge of the gear are set to 150, which means they are a non-inspection area. Black, white and some other gray colors indicate different parts to be detected.

The templates are collected from a standard and perfect gear. Here is the acquisition process of top surface template. First extract the gear’s center and rotation angle, and then divide target pixels into different parts according to their distance to the center and mark with different colors, remove background pixels completely. The templates are numbered and saved in order of rotation angle.

**Figure 16:**
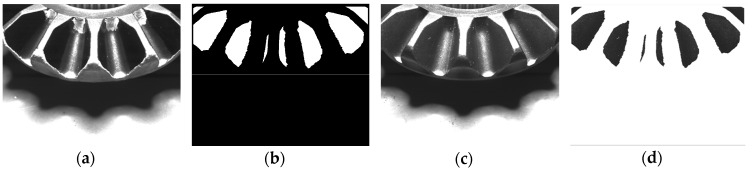
The collection and use of bevel gear lateral template. (**a**) Lateral image of template gear; (**b**) Extracted template; (**c**) Lateral image to be detected; (**d**) Extracted image by template matching.

The lateral template is collected by the aid of a special gear painted with black and white matted coating, shown in Figure 16a. This ensures that a perfect template can be extracted through a simple image processing, shown in Figure 16b. Figure 16c is an original lateral image and Figure 16d is the extracted teeth surface by template matching.

In this system the templates collection and processing are completed automatically without artificial participation. Templates are automatically numbered and saved in order of rotation angle and camera. After a template is collected, the gear is rotated to another angle by the step-motor and the next collection begins. The angle interval is about 0.01°–0.02°. The total template image size is as large as 25 GB, that is to say, the system achieves ideal inspection speed by sacrificing storage space.

## 4. Inspection Results

**Figure 17:**
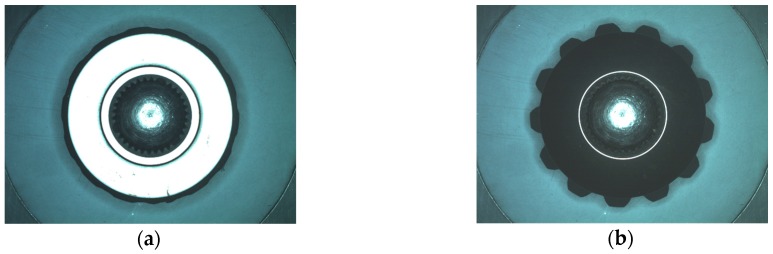
Bottom images of F_6_ and A_4_: (**a**) Bottom of F_6_; (**b**) Bottom of A_4_.

The inspection tasks and methods are different for bevel gears vary in structure. Figure 17a,b are bottom surfaces of gear F_6_ and A_4_ respectively. F_6_ is flat and defects are obvious in the image. It is easy to detect. A_4_ is spherical and the image is dark and weak in contrast. The defects are hard to be found in the image, especially for dints. The inspection algorithm of A_4_ is much more complicated.

### 4.1. Example of Defect Detection and Dimension Measurement

In this section, we illustrate how the system works through a practical example, the inspection of bottom surface of bevel gear A_4_.

#### 4.1.1. Inspection of Top-Circle on Bottom Surface

**Figure 18:**
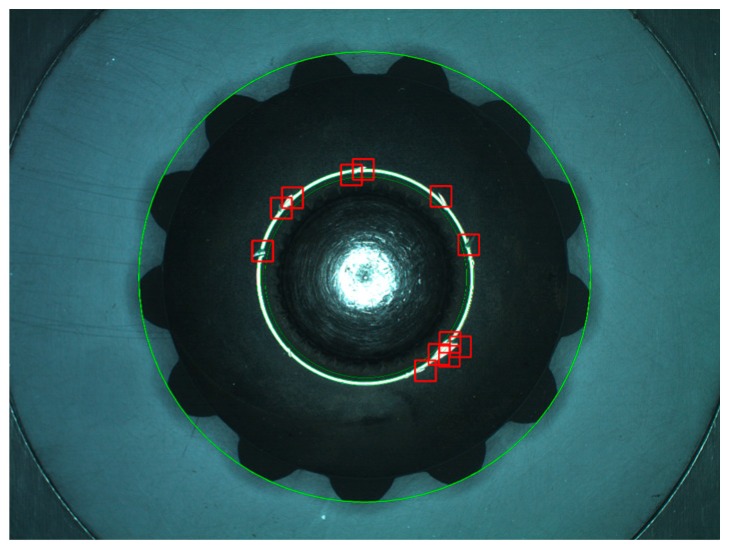
Inspection of bottom surface of gear A_4_, red boxes mark defects and green circle marks circumcircle.

The top of back surface is a standard sharp ring in the image of a perfect A_4_ gear. But it is fragile and easily to be damaged in production process. Defects are shown as broken ring, burr or uneven color in the image. The system will detect and mark the defects as shown in Figure 18. The following illustrate the detail inspection process.

**Figure 19:**
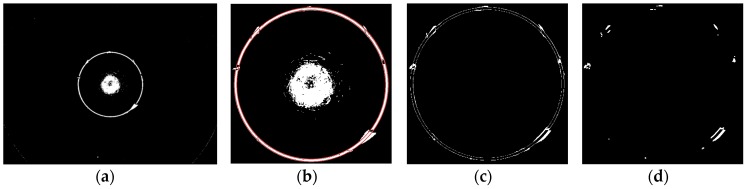
Defect detection of top ring on bottom surface: (**a**) Top ring extraction; (**b**) Top ring fitting (red circles); (**c**) Result of defect detection; (**d**) Defects after open-operation.

*Step 1*: Extract the ring and its neighborhood by threshold segmentation, and then remove small noise around the ring by area filter, the result is shown in Figure 19a.
*Step 2*: Fitting the ring boundaries. Generally the bevel gear is centrosymmetric, figure out the approximate center by average coordinate. Fit the inner and outer boundary circles of the ring by CAM. Red circles in Figure 19b are the fitting result. This is preparatory work for Step 3 and the following dimension measurement.
*Step 3*: Defect detection. The pixels of the top ring are highlighted for a qualified gear, so the dark pixels between two red circles are marked as defects. For the inside and outside neighborhood of the ring, defects are located by the combination of fixed and dynamic threshold segmentation. The merged result is shown as the white pixel groups in Figure 19c. Then eliminate additional arc by image open-operation and extract bigger defects by area filtering, as shown in Figure 19d. The end result is marked by red boxes in Figure 18.

#### 4.1.2. Inspection of Spherical Surface

**Figure 20:**
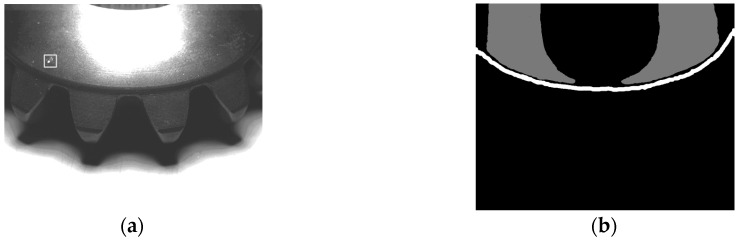
(**a**) The lateral image of sphere surface; (**b**) The template of sphere surface.

The defects on spherical surface are not obvious in the top camera image and should be detected from lateral camera images. Figure 20a shows such an image. The region just facing the camera is highly lit and both sides are darker and darker. Experiments show that defects in the transition region are the most obvious, so the main inspection area is between the high light and the darkest regions.

To extract the target region fast, the system creates a template for each lateral camera, as shown in Figure 20b. Gray indicates the inspection region and black/white is the non-inspection region. The white stripe is used for device calibration and avoiding erroneous inspections. The whole sphere surface cannot be covered by seven cameras at a time. To ensure the defects can be detected at any angle, the gear is rotated 3 to 5 times by a stepper motor.

**Figure 21:**
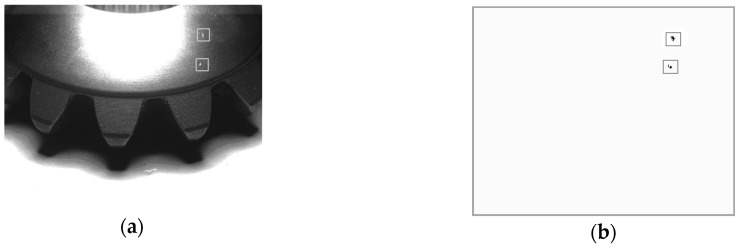
(**a**) A lateral image of the sphere surface; (**b**) Inspection result of Figure 21a.

The motor rotation is time-consuming. The motor begins to rotate just at the end of a single image acquisition. The rotation completes when the image processing finishes, then the next image acquisition begins. Inspection time is saved greatly by this process. The defects are detected by the NAD method. Figure 21a shows an image with defects in it and Figure 21b shows the inspection result.

#### 4.1.3. Measurement of End Rabbet Diameter

**Figure 22:**
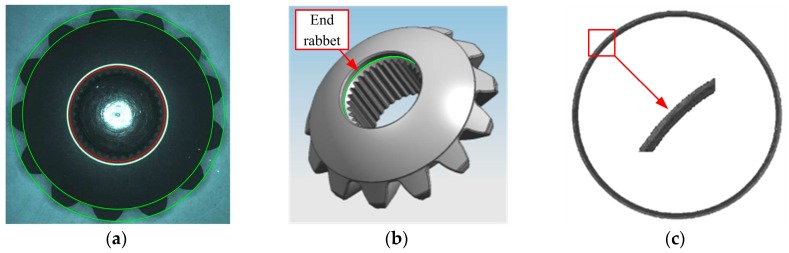
Size measurement of circumcircles and end rabbet: (**a**) Green circles are circumcircles and red is end rabbet circle. (**b**) Location of end rabbet; (**c**) Extracted rabbet and its amplified image.

Size measurement includes the diameters of large and small circumcircles and end rabbets. The two circumcircles are relatively easy to fit by threshold segmentation and CAM. The fitting results are shown as green circles in Figure 22a. The end rabbet is a shallow circular groove on the gear. Figure 22b shows its location and 22c is an amplified image. The division of two regions of different gray values in Figure 22c is the rabbet circle. It is dark in the image and hard to identify.

**Figure 23:**
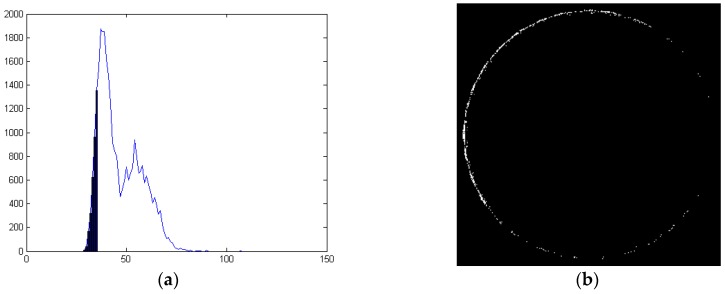
(**a**) Histogram of End rabbet and nearby pixels; (**b**) Extract the outline of rabbet circle.

Because the rabbet circle is not distinct in the image, fixed or dynamic thresholding such as the Otsu algorithm or the interactive method are not applicable [20,21,22]. Figure 23a shows the gray histogram of the rabbet circle and its nearby pixels. We find that the pixels on the rabbet circle are the darkest. They are the left part of the histogram, about 3000–5000 pixels. We extract these pixels and then get the outline of the rabbet circle, as shown in Figure 23b.

We use CAM to fit the rabbet circle in Figure 23b. The result is shown as the red circle in Figure 22a. We prove again that our method is much faster than the Hough Transform and least-squares method while ensuring the accuracy.

### 4.2. Statistical Results of Bevel Gear Inspection

Using the inspection system developed in this paper, 84 bevel gears of type A_4_ and 84 of type F_6_ are tested. Table 2, Table 3 and Table 4 are the inspection results of A_4_. Table 5, Table 6 and Table 7 are the inspection results of F_6_. The data shows that size measurement of our system is 100% accurate under the current requirements. Measurement accuracy is about 45 μm and repeated error is no more than 90 μm. The minimum detectable defect is 0.4 mm × 0.4 mm. Inspection time of one surface is less than 1.3 s. Both the accuracy and the speed are suitable for the real-time on-line inspection in automatic production lines.

## 5. Discussion

The presented multi-camera automobile bevel gear quality inspection system adopts advanced machine vision technology. It solves the problem of difficult inspection of complicated gears at high speed and with high precision. The research shows that machine vision can substitute most manual work in bevel gear inspection, improving the efficiency of production and the degree of automation.

The prototype of this system has been realized and applied in bevel gear production. There are still some technical problems to be solved. The edge of the gear is a non-detectable area in our system and some defects in low contrast images cannot be detected reliably. We are seeking better illumination schemes and new inspection ideas to solve these problems.

## Figures and Tables

**Table 1:** Compare Circle Approximation Method with Hough Transform in moon fitting.

Method	Min Radius/pixel	Max Radius/pixel	Center	Step Size of Angle/rad	Step Aize of Radius/pixel	Time/s
Hough Transform	150	250	Null	0.1	1	5.6623
CAM	150	250	Image center	0.006	0.5	0.1189

**Table 2:** Top surface inspection of gear A_4_.

Results	Top Circle		Teeth Surface			Teeth Edge	
	Connected Fault	Knock	Dent	Knock	Insufficient Filling	Knock	Insufficient Filling
Number of defects	1	7	10	15	1	13	10
Detected defects	1	7	10	12	1	11	10
Accuracy/%	100	100	100	80	100	84.6	100

**Table 3:** Lateral surface inspection of gear A_4_.

Results	Knock	Scratch	Dent	Gibbosity	Roughness	Peeling off
Number of defects	30	23	18	1	6	1
Detected defects	28	19	16	1	6	1
Accuracy/%	93.3	82.6	88.9	100	100	100

**Table 4:** Bottom surface inspection of gear A_4_.

Results	Bottom Ring		Spherical Surface			Spline
	Knock	Scratch	Knock	Scratch	Dent	Duplicated Broaching
Number of defects	41	12	15	23	19	6
Detected defects	39	12	15	22	19	6
Accuracy/%	95.1	100	100	95.6	100	100

**Table 5:** Top surface inspection of gear F_6_.

Results	Top Ring			Top Teeth Surface		Teeth Edge	
	Chamfer Lack	Knock	Dent	Knock	Dent	Knock	Insufficient Filling
Number of defects	2	7	8	17	9	34	18
Detected defects	2	7	8	16	9	30	18
Accuracy/%	100	100	100	94.1	100	88.2	100

**Table 6:** Lateral surface inspection of gear F_6_.

Results	Lateral Teeth Surface				Lateral Teeth Small Surface		
	Knock	Dent	Scratch	Insufficient Filling	Knock	Dent	Scratch
Number of defects	42	8	11	3	13	5	4
Detected defects	37	8	8	3	11	5	3
Accuracy/%	88.1	100	72.7	100	84.6	100	75

**Table 7:** Bottom surface inspection of gear F_6_.

Results	Bottom Ring		Mounting Surface			Spline
	Knock	Rough	Knock	Scratch	Size Error	Duplicated Broaching
Number of defects	3	3	2	5	4	3
Detected defects	3	3	2	4	4	3
Accuracy/%	100	100	100	80	100	100

## Abbreviations

NAD	Neighborhood Average Difference
CAM	Circle Approximation Method
FRP	Fast Rotation-Position
